# Dirac Equation and Fisher Information

**DOI:** 10.3390/e26110971

**Published:** 2024-11-12

**Authors:** Asher Yahalom

**Affiliations:** 1 Department of Electrical & Electronic Engineering, Faculty of Engineering, Ariel University, Ariel 40700, Israel; asya@ariel.ac.il; Tel.: +972-54-7740294; 2 Center for Astrophysics, Geophysics, and Space Sciences (AGASS), Ariel University, Ariel 40700, Israel; 3 FEL User Center, Ariel University, Ariel 40700, Israel

**Keywords:** spin, relativistic fluid dynamics, relativistic quantum mechanics

## Abstract

Previously, it was shown that Schrödinger’s theory can be derived from a potential flow Lagrangian provided a Fisher information term is added. This approach was later expanded to Pauli’s theory of an electron with spin, which required a Clebsch flow Lagrangian with non-zero vorticity. Here, we use the recent relativistic flow Lagrangian to represent Dirac’s theory with the addition of a Lorentz invariant Fisher information term as is required by quantum mechanics.

## 1. Introduction

Quantum mechanics is commonly understood through the Copenhagen interpretation, which views the quantum wave function as an epistemological tool, used solely for predicting measurement probabilities, aligning with Kantian philosophy that denies human ability to understand things “as they are” (ontology) [1]. However, another prominent school of thought interprets quantum mechanics differently, believing in the wave function’s reality. This view, supported by Einstein and Bohm [2,3,4], considers the wave function as a real entity akin to an electromagnetic field. This perspective led to alternative interpretations, such as Madelung’s fluid realization [5], which proposes that the square of the wave function’s modulus represents a fluid density, and its phase represents the fluid’s velocity potential. However, this approach is limited to spinless electron wave functions and cannot account for a full set of attributes even for slow-moving electrons.

Wolfgang Pauli [6] introduced a non-relativistic quantum equation for a spinor in 1927. This equation utilizes a two-dimensional operator matrix Hamiltonian. It was shown that such a theory can be interpreted through fluid dynamics [7]. This interpretation is significant because proponents of the Copenhagen interpretation of quantum mechanics often use the concept of spin as evidence that nature has inherently quantum elements without classical analogues or interpretations.

Holland [3] and others provided a Bohmian analysis of the Pauli equation, but they did not address the analogy between Pauli theory and fluid dynamics or the notion of spin vorticity. Thus, spin fluid dynamics for a single electron with spin [7] was introduced subsequently.

Interpreting Pauli’s spinor in terms of fluid density and velocity variables connects us to the 19th-century work of Clebsch, which is closely tied to the Eulerian variational analysis of fluids. Clebsch [8,9] and, much later, Davidov [10] described variational principles for barotropic fluid dynamics. Clebsch introduced a four-function variational principle for an Eulerian barotropic fluid, and Davidov aimed to quantize fluid dynamics, though his work was not well known in the West due to it being written in Russian. Eckart [11] provided a variational description for Lagrangian fluid dynamics, which differs from the variational approach to Eulerian fluid dynamics.

Initial attempts to formulate Eulerian fluid dynamics using variational principles in the English literature were made by Herivel [12], Serrin [13], and Lin [14]. However, their methods were complicated, relying on numerous Lagrange multipliers and auxiliary potentials, involving between eleven and seven independent functions—more than the four required for the Eulerian and continuity equations of barotropic flow, making these methods impractical.

Seliger and Whitham [15] reintroduced Clebsch’s variational formalism using only four variables for barotropic flow. Lynden-Bell and Katz [16] proposed a variational principle in terms of two functions, load λ and density ρ, but their approach had an implicit definition of velocity $\vec{v}$, requiring the solution of a partial differential equation to determine $\vec{v}$ in terms of ρ and λ and its variations. Yahalom and Lynden-Bell [17] addressed this limitation by adding one more variational variable, allowing for arbitrary (unconstrained) variations and providing an explicit definition of $\vec{v}$.

A key challenge in interpreting quantum mechanics through fluid dynamics lies in understanding thermodynamic quantities. In traditional fluids, concepts like specific enthalpy, pressure, and temperature relate to specific internal energy, which is a unique function of entropy and density defined by the equation of state. This internal energy can be explained through the microscopic composition of the fluid using statistical physics, based on the interactions of atoms, ions, electrons, and molecules through electromagnetic fields.

However, a quantum fluid lacks such structure. Yet, equations for both spinless [5,7] and spin [7] quantum fluid dynamics show terms analogous to internal energies. This raises the question: where do these internal energies come from? Suggesting that quantum fluids have a microscopic substructure contradicts empirical evidence that electrons are point particles.

The answer lies in measurement theory, particularly Fisher information [18,19], a measure of the quality of any quantity’s measurement. It has been shown [19] that Fisher information corresponds to the internal energy of a non-relativistic spinless electron (up to a proportionality constant) and can partly explain the internal energy of a non-relativistic electron with spin [19].

There has been an attempt to derive most physical theories from Fisher information by Frieden [20]; however, in this approach there is always a *J* component to the Lagrangian (in addition to the Fisher information) which is unique to each physical system and is chosen without justification such that the desired Lagrangian is derived.

At the time of Clebsch, relativity was not introduced yet; hence, there was no need to write a variational principle for an Eulerian relativistic flow (which was an invariant under a Lorentz transformation). This was recently rectified in [21] in which relativistic Clebsch fluid dynamics was introduced. It was shown also that relativistic Clebsch fluid dynamics can lead to relativistic quantum mechanics by adding a Lorentz invariant Fisher information term. For null vorticity and low velocities, this variational principle reduces to the Schrödinger variational principle.

Thus, it is now needed to compare the fluid-derived relativistic quantum mechanics to the more prevalent Dirac theory, which is the current established theory of relativistic quantum mechanics. This comparison involves several steps, the first of which is to express the theory in terms of four variables of a fluid (velocity vector and density) rather than the eight (a complex four spinor) of Dirac. This will be conducted in the following sections.

## 2. Dirac Theory

The theory of Dirac is defined in terms of the equation (we initially neglect the electromagnetic interactions):

(1) $$\left(iћ\gamma^{\mu }\partial_{\mu }-mc\right)\Psi =0$$

where Ψ is a four dimensional complex column vector (spinor), and $\gamma^{\mu }$ are four-dimensional complex matrices satisfying the anticommutation relations:

(2) $$\left\{\gamma^{\mu },\gamma^{\nu }\right\}=2\eta^{\mu \nu }I_{4},\;\eta^{\mu \nu }=\mathrm{diag}\left(+1,-1,-1,-1\right)$$

where $I_{4}$ is a unit matrix in four dimensions. In what follows, Greek indices are μ,ν∈{0,1,2,3} and Latin indices are i,j,k∈{1,2,3}. There are multiple representations of $\gamma^{\nu }$; we shall use the following representation:

(3) $$\gamma^{0}=\left(\begin{array}{cc} I_{2} & 0\\ 0 & -I_{2} \end{array}\right)\;\gamma^{i}=\left(\begin{array}{cc} 0 & \sigma^{i}\\ -\sigma^{i} & 0 \end{array}\right)$$

(4) $$\sigma^{1}=\left(\begin{array}{cc} 0 & 1\\ 1 & 0 \end{array}\right),\;\sigma^{2}=\left(\begin{array}{cc} 0 & -i\\ i & 0 \end{array}\right),\;\sigma^{3}=\left(\begin{array}{cc} 1 & 0\\ 0 & -1 \end{array}\right).$$

and $I_{2}$ is a unit matrix in two dimensions. Equation (1) may be integrated provided initial conditions are supplied; that is:

(5) $$\Psi \left(0,\vec{x}\right)=\Psi_{0}\left(\vec{x}\right)$$

The theory seems disconnected from any fluid dynamic interpretation as Ψ depends on eight scalar quantities, while a barotropic fluid theory depends on only four variables (half the required amount). However, the theory can be expressed in terms of fewer variables as follows. First, we write the four-dimensional spinor in terms of two-dimensional spinors:

(6) $$\Psi =\left(\begin{array}{c} \psi_{1}\\ \psi_{2} \end{array}\right).$$

This form induces by Equation (5) the initial condition on both $\psi_{1}$ and $\psi_{2}$:

(7) $$\psi_{1}\left(0,\vec{x}\right)=\psi_{10}\left(\vec{x}\right),\;\psi_{2}\left(0,\vec{x}\right)=\psi_{20}\left(\vec{x}\right),\;\Psi_{0}=\left(\begin{array}{c} \psi_{10}\\ \psi_{20} \end{array}\right).$$

Substituting Equation (6) in Equation (1) we obtain:

(8) $$\left(iћ\partial_{0}-mc\right)\psi_{1}+iћ\sigma^{i}\partial_{i}\psi_{2}=0,\;\left(iћ\partial_{0}+mc\right)\psi_{2}+iћ\sigma^{i}\partial_{i}\psi_{1}=0$$

introducing the hatted variables:

(9) $${\hat{\psi }}_{1}\equiv e^{-\frac{imc}{ћ}x_{0}}\psi_{1}=e^{-\frac{imc^{2}}{ћ}t}\psi_{1},\;{\hat{\psi }}_{2}\equiv e^{-\frac{imc}{ћ}x_{0}}\psi_{2}=e^{-\frac{imc^{2}}{ћ}t}\psi_{2}.$$

We can substitute:

(10) $$\psi_{1}=e^{+\frac{imc}{ћ}x_{0}}{\hat{\psi }}_{1},\;\psi_{2}=e^{+\frac{imc}{ћ}x_{0}}{\hat{\psi }}_{2}.$$

in Equation (8) and obtain the simplified set of equations:

(11) $$\left(iћ\partial_{0}-2mc\right){\hat{\psi }}_{1}+iћ\sigma^{i}\partial_{i}{\hat{\psi }}_{2}=0,\;iћ\partial_{0}{\hat{\psi }}_{2}+iћ\sigma^{i}\partial_{i}{\hat{\psi }}_{1}=0$$

The initial conditions for these equations at $x_{0}=0$ are the same as before because ψ and $\hat{\psi }$ are the same at that particular time:

(12) $${\hat{\psi }}_{1}\left(0,\vec{x}\right)=\psi_{10}\left(\vec{x}\right),\;{\hat{\psi }}_{2}\left(0,\vec{x}\right)=\psi_{20}\left(\vec{x}\right).$$

The second equation for ${\hat{\psi }}_{2}$ can be readily solved if ${\hat{\psi }}_{1}$ is known:

(13) $${\hat{\psi }}_{2}\left(x_{0},\vec{x}\right)\left[{\hat{\psi }}_{1}\right]={\hat{\psi }}_{2}\left(0,\vec{x}\right)-\sigma^{i}\partial_{i}\int_{0}^{x_{0}}{\hat{\psi }}_{1}\left(x_{0}^{\prime },\vec{x}\right)dx_{0}^{\prime }$$

Introducing the auxiliary variable:

(14) $$int{\hat{\psi }}_{1}\equiv \int_{0}^{x_{0}}{\hat{\psi }}_{1}\left(x_{0}^{\prime },\vec{x}\right)dx_{0}^{\prime }\Rightarrow \partial_{0}int{\hat{\psi }}_{1}={\hat{\psi }}_{1},\partial_{0}^{2}int{\hat{\psi }}_{1}=\partial_{0}{\hat{\psi }}_{1}$$

and the time-independent spinor:

(15) $$W\left(\vec{x}\right)\equiv -\sigma^{k}\partial_{k}{\hat{\psi }}_{2}\left(0,\vec{x}\right),$$

we may write Equation (11), and thus the Dirac theory, in the form:

(16) $$\left(\partial^{\mu }\partial_{\mu }+2\frac{imc}{ћ}\partial_{0}\right)int{\hat{\psi }}_{1}=W\left(\vec{x}\right),\;{\hat{\psi }}_{2}\left(x_{0},\vec{x}\right)={\hat{\psi }}_{2}\left(0,\vec{x}\right)-\sigma^{i}\partial_{i}int{\hat{\psi }}_{1}$$

Hence, the mathematical problem of Dirac theory is to solve the first part of Equation (16) because the second equation is just a relation giving us ${\hat{\psi }}_{2}$ explicitly in terms of ${\hat{\psi }}_{1}$. Moreover, if we have a solution for ${\hat{\psi }}_{1}$, then $int{\hat{\psi }}_{1}$ follows immediately from Equation (14). Taking the temporal partial derivative of the first equation in (16), it follows that ${\hat{\psi }}_{1}$ must satisfy the equation:

(17) $$\left(\partial^{\mu }\partial_{\mu }+2\frac{imc}{ћ}\partial_{0}\right){\hat{\psi }}_{1}=0$$

The initial conditions of this second-order equation are fixed by the initial conditions of Equation (11) because those conditions also fix the first derivative in time $x_{0}=0$.

(18) $$\left(iћ\partial_{0}-2mc\right){\hat{\psi }}_{1}{{|}_{x_{0}=0}+iћ\sigma^{i}\partial_{i}\psi_{20}=0,\;\partial_{0}{\hat{\psi }}_{2}|}_{x_{0}=0}+\sigma^{i}\partial_{i}\psi_{10}=0.$$

Or more simply as:

(19) $$\partial_{0}{\hat{\psi }}_{1}{|}_{x_{0}=0}=\frac{2mc}{iћ}\psi_{10}-\sigma^{i}\partial_{i}\psi_{20}$$

As we are given both the initial condition of the function and the initial condition of its first derivative, the solution of the second-order differential Equation (17) is fixed, and its solution is the entire content of the Dirac theory. We notice at this point that one can reintroduce the original function $\psi_{1}$ using Equation (9), which will result in the Klein–Gordon equation:

(20) $$\left(\partial^{\mu }\partial_{\mu }+\frac{m^{2}c^{2}}{{ћ}^{2}}\right)\psi_{1}=0$$

with the initial conditions:

(21) $$\psi_{1}{{|}_{x_{0}=0}=\psi_{10},\;\partial_{0}\psi_{1}|}_{x_{0}=0}=\frac{mc}{iћ}\psi_{10}-\sigma^{i}\partial_{i}\psi_{20}.$$

which is also equivalent to Dirac’s theory. However, notice first that in this case, the Klein–Gordon equation is an equation for a two-dimensional spinor not a scalar or even a complex scalar. Second, the physical interpretation in Dirac theory is quite different with respect to the original Klein–Gordon theory. In particular, the conserved probability four current is:

(22) $$J^{\mu }\equiv \bar{\Psi }\gamma^{\mu }\Psi ,\;\bar{\Psi }\equiv \Psi^{†}\gamma^{0}$$

Thus, we obtain the probability density:

(23) $$J^{0}=\bar{\Psi }\gamma^{0}\Psi =\Psi^{†}{\left(\gamma^{0}\right)}^{2}\Psi =\Psi^{†}\Psi =\psi_{1}^{†}\psi_{1}+\psi_{2}^{†}\psi_{2}\geq 0.$$

This is quite different from $J^{0}$ in the original Klein–Gordon theory, which could become negative, and thus, unphysical. Nevertheless, we have shown that from a mathematical point of view, both theories have identical equations but different mathematical dependent variables. In the Klein–Gordon theory, we consider (complex) scalars, and in the Dirac theory, we consider spinors. We are in a better position now to show the analogies with relativistic flows as at least both theories depend on an identical number of dependent variables that is four scalar functions.

## 3. Variational Description

Equation (20) can be deduced from a variational principle using the Lagrangian density:

(24) $$L_{KG}\equiv m\left(\frac{{ћ}^{2}}{m^{2}}\partial^{\mu }\psi_{1}^{†}\partial_{\mu }\psi_{1}-c^{2}\psi_{1}^{†}\psi_{1}\right),\;A_{KG}\equiv \int d^{4}xL_{KG}$$

provided that the variations are constrained in a suitable manner on the spatial and temporal boundaries. This is not the traditional Dirac Lagrangian density but has the same mathematical content nonetheless, as we showed in the previous section. Let us write the two-dimensional spinor $\psi_{1}$ in terms of its up and down components:

(25) $$\psi_{1}=\left(\begin{array}{c} \psi_{↑}\\ \psi_{↓} \end{array}\right).$$

Inserting Equation (25) into Equation (24), we obtain:

(26) $$L_{KG}=m\left(\frac{{ћ}^{2}}{m^{2}}\partial^{\mu }\psi_{↑}^{*}\partial_{\mu }\psi_{↑}-c^{2}\psi_{↑}^{*}\psi_{↑}+\frac{{ћ}^{2}}{m^{2}}\partial^{\mu }\psi_{↓}^{*}\partial_{\mu }\psi_{↓}-c^{2}\psi_{↓}^{*}\psi_{↓}\right).$$

We now write the up and down wave functions in an amplitude and phase representation:

(27) $$\psi_{↑}=R_{↑}e^{i\frac{m}{ћ}\nu_{↑}},\;\psi_{↓}=R_{↓}e^{i\frac{m}{ћ}\nu_{↓}}$$

Substituting Equation (27) into Equation (26) will lead to the form:

(28) $$\begin{array}{ccc} L_{KG} & = & L_{KGq}+L_{KGc}\\ L_{KGq} & \equiv & \frac{{ћ}^{2}}{m}\left(\partial^{\mu }R_{↑}\partial_{\mu }R_{↑}+\partial^{\mu }R_{↓}\partial_{\mu }R_{↓}\right)\\ L_{KGc} & \equiv & m\left(R_{↑}^{2}\left(\partial^{\mu }\nu_{↑}\partial_{\mu }\nu_{↑}-c^{2}\right)+R_{↓}^{2}\left(\partial^{\mu }\nu_{↓}\partial_{\mu }\nu_{↓}-c^{2}\right)\right). \end{array}$$

in which we have partitioned $L_{KG}$ into a quantum part $L_{KGq}$ and a classical part $L_{KGc}$. In the classical limit in which ћ→0:

(29) $$\lim_{ћ\to 0}L_{KGq}=0\;\Rightarrow \;\lim_{ћ\to 0}L_{KG}=L_{KGc}$$

We introduce a mass density and an angle θ in the following natural way:

(30) $$\bar{\rho }\equiv m\left(R_{↑}^{2}+R_{↓}^{2}\right),\;\tan\theta \equiv \frac{R_{↓}}{R_{↑}},\;\Rightarrow \;R_{↑}=\sqrt{\frac{\bar{\rho }}{m}}\cos\theta ,\;R_{↓}=\sqrt{\frac{\bar{\rho }}{m}}\sin\theta$$

It thus follows that:

(31) $$L_{KGc}=\bar{\rho }\left[\cos^{2}\theta \partial^{\mu }\nu_{↑}\partial_{\mu }\nu_{↑}+\sin^{2}\theta \partial^{\mu }\nu_{↓}\partial_{\mu }\nu_{↓}-c^{2}\right].$$

Now, let us set:

(32) $$\begin{array}{ccc} \nu & \equiv & \nu_{↑},\;\beta \equiv \nu_{↓}-\nu_{↑},\\ \alpha & \equiv & \frac{-\partial_{\mu }\nu \partial^{\mu }\beta \pm \sqrt{{\left(\partial_{\mu }\nu \partial^{\mu }\beta \right)}^{2}+\sin^{2}\theta \left(\partial_{\mu }\beta \partial^{\mu }\beta \right)\left(\partial^{\mu }\beta \left(2\partial_{\mu }\nu +\partial_{\mu }\beta \right)\right)}}{\partial_{\mu }\beta \partial^{\mu }\beta } \end{array}$$

in terms of which we define a four-dimensional Clebsch field:

(33) $$v_{C}^{\mu }\equiv \alpha \partial^{\mu }\beta +\partial^{\mu }\nu$$

Plugging Equation (33) and using the definitions of Equation (32), we obtain after some cumbersome but straightforward calculations the result:

(34) $$L_{KGc}=\bar{\rho }\left[v_{C}^{\mu }v_{C\mu }-c^{2}\right].$$

Defining the mass density in the rest frame as:

(35) $$\rho_{0}=\frac{\bar{\rho }}{c}\left[\sqrt{v_{C}^{\mu }v_{C\mu }}+c\right].$$

it follows that:

(36) $$L_{KGc}=c\rho_{0}\left[\sqrt{v_{C}^{\mu }v_{C\mu }}-c\right]=L_{\mathrm{Relativistic}\;\mathrm{Flow}}.$$

Thus, the classical part of $L_{KG}$ is equivalent (although in a non-trivial way) to a Lagrangian density of a classical relativistic fluid but, of course, without an internal energy (see Equation (103) in [21]). We note that unlike the non-relativistic Pauli spin flow, which has a classical redundant term (in the sense that it does not comply with the fluid frame work) of the form (see Equation (63) in [19]):

(37) $$\lim_{ћ\to 0}\varepsilon_{qs}=\frac{1}{2}\left(1-\alpha^{2}\right){\left(\vec{\nabla }\beta \right)}^{2}$$

in Dirac theory, we have a perfect mapping between the classical parts of Dirac’s Lagrangian and the relativistic flows, without any “left overs”.

## 4. The Dirac Quantum Term

Let us now compare the Dirac quantum term $L_{KGq}$ appearing in Equation (28) to the quantum Fisher information term appearing in Equation (113) of [21], which we cite here for completeness:

(38) $$L_{RFq}=\frac{{ћ}^{2}}{2m}\partial^{\mu }a_{0}\partial_{\mu }a_{0},\;a_{0}\equiv \sqrt{\frac{\rho_{0}}{m}}.$$

On a superficial consideration, they look quite the same; however, looking more closely, striking differences appear. First, $L_{KGq}$ depends on two “density amplitudes” (one for each spin) as opposed to the single amplitude of $L_{RFq}$. Indeed, it is known that each energy eigenstate of the Dirac equation can accommodate two electrons each with a different spin. Second, a factor of 2 is missing in the denominator of $L_{KGq}$. We shall try to answer the questions in the following. Looking at Equation (35), we recall that the amplitudes $R_{↑}$ and $R_{↓}$ are not simply connected to the density as:

(39) $$\rho_{0}=\bar{\rho }\left[\sqrt{\frac{v_{C}^{\mu }v_{C\mu }}{c^{2}}}+1\right].$$

However, according to Equation (104) of [21]:

(40) $$\sqrt{v_{C\mu }v_{C}^{\mu }}=|v_{C0}|\sqrt{1-\frac{{\vec{v}}_{C}^{2}}{v_{C0}^{2}}}=\left|v_{C0}\right|\sqrt{1-\frac{{\vec{v}}^{2}}{c^{2}}}=\frac{|v_{C0}|}{\gamma }$$

Moreover, according to Equation (101) of [21]:

(41) $$|v_{C0}|=c\lambda$$

For a classical fluid lacking internal energy and satisfying the equations of motion (see Equation (58) of [21]):

(42) λ=γ

thus up to quantum corrections:

(43) $$\sqrt{v_{C\mu }v_{C}^{\mu }}\approx c$$

Inserting Equation (43) into Equation (39)

(44) $$\rho_{0}\approx 2\bar{\rho }.$$

Thus:

(45) $$a_{0}=\sqrt{\frac{\rho_{0}}{m}}\approx \sqrt{\frac{2\bar{\rho }}{m}}=\sqrt{2}R,\;R^{2}\equiv R_{↓}^{2}+R_{↑}^{2}$$

In terms of *R* and θ, one may write the quantum part of the Lagrangian density as:

(46) $$L_{KGq}=\frac{{ћ}^{2}}{m}\left(\partial^{\mu }R_{↑}\partial_{\mu }R_{↑}+\partial^{\mu }R_{↓}\partial_{\mu }R_{↓}\right)=\frac{{ћ}^{2}}{m}\left(\partial^{\mu }R\partial_{\mu }R+R^{2}\partial^{\mu }\theta \partial_{\mu }\theta \right).$$

Thus:

(47) $$L_{KGq}\approx \frac{{ћ}^{2}}{2m}\left(\partial^{\mu }a_{0}\partial_{\mu }a_{0}+a_{0}^{2}\partial^{\mu }\theta \partial_{\mu }\theta \right).$$

We notice that in Dirac’s theory, *R* is not a probability amplitude as according to Equation (23)

(48) $$J^{0}=\psi_{1}^{†}\psi_{1}+\psi_{2}^{†}\psi_{2}=R^{2}+\psi_{2}^{†}\psi_{2}\geq R^{2}.$$

Thus, the second term in the quantum Lagrangian density perhaps is not surprising, and of course, a complete calculation requires the inclusion of the quantum effects neglected in Equation (43).

## 5. Conclusions

We have shown the equivalence of the classic sector of Dirac theory to relativistic fluid dynamics. This solves the riddle about some strange terms appearing in the fluid description of Pauli’s theory. However, the quantum sector of Dirac’s theory contains an additional term (the same “redundant” terms appear also in the fluid representation of Pauli’s theory [19]), which is not expected based on purely Fisher information considerations. Thus, a deeper study is warranted, taking into account both quantum contributions to the λ term, which is a property of the relativistic fluid, and also the unique definition of the probability density in Dirac’s theory, taking into account all four spinor amplitudes. This important task is left for the future.

In a series of papers, it was shown that there is a new route to quantization which is neither canonical quantization (through postulating commutation relations) nor path integral, and this is the Fisher information approach [19,21]. This approach seems to work in both relativistic and non-relativistic quantum mechanics. Hence, one cannot be surprised that the Fisher information terms appear in Dirac theory as the accepted relativistic quantum mechanics formalism; this is hardly a mathematical coincidence.

The current work may be related to the event-based interpretation of quantum mechanics in which events are taken as primitive notions (as customary in relativity), whereas quantum systems (e.g., fields and particles) are emergent in the form of joint probability amplitudes for position and time of events [22,23,24]. The appearance of an information-related term in this context is perhaps not all that surprising since such a term might have some utility in calculating a probability.

Of course, a complete description of the physics of the electron in terms of Dirac theory will require the interaction of the electron with the electromagnetic field, which implies four potential terms in the variational action. This is also left for future endeavours.

Finally, we remark that the nature of the quantum relativistic flow remains quite mysterious. One cannot avoid the obvious question: “a flow of what?” This fundamental question has consequences for both the issues raised above (the strange Fisher information addition and the existence of electromagnetic fields). We offer a rather bold hypothesis that dates back to Riemann regarding the geometry of space-time. According to Riemann [25], all physical entities are geometrical; hence, the flow is just the geometrical description of some thin elongated defect in space-time (thin in spatial dimensions but elongated in the temporal direction). The position of this defect is not well defined, which is the reason for the appearance of the Fisher information term. We recall that, based on Riemann’s proposal, Einstein suggested the very successful theory of general relativity [26] describing gravity to high precision as the metric of space-time. However, an attempt of Weyl [25] to geometrize the electromagnetic field based on affine geometry is regarded as less successful. Moreover, the idea of Schrödinger [27] to geometrize matter based on the non-symmetric affine connection is not considered successful. Yet, we are hopeful that the current mapping of relativistic flow to Dirac theory may shed some light on those early attempts and some progress can be made.

Thus, the ontological significance of either the classical or the quantum parts of the Lagrangian density is indeed an open question. While the flow may (or may not) be related to the additional geometrical structure as suggested by Schrödinger (see comment above), the quantum part, which is essentially a Fisher information term, may be related to the fact that we enforce on the nature the mathematical structure of a smooth manifold where, in reality, it is a connected network of discrete nodes; more on this can be found in [28] and references therein.

## Data Availability

No new data were created or analyzed in this study. Data sharing is not applicable to this article.
